# Effects of Organic Acid Root Exudates of *Malus hupehensis* Rehd. Derived from Soil and Root Leaching Liquor from Orchards with Apple Replant Disease

**DOI:** 10.3390/plants11212968

**Published:** 2022-11-03

**Authors:** Nan Sun, Chen Yang, Xin Qin, Yangbo Liu, Mengyi Sui, Yawen Zhang, Xueli Cui, Yijun Yin, Rong Wang, Yanli Hu, Xuesen Chen, Zhiquan Mao, Yunfei Mao, Xiang Shen

**Affiliations:** 1 State Key Laboratory of Crop Biology, College of Horticulture Science and Engineering, Shandong Agricultural University, Tai’an 271002, China; 2 College of Horticulture and Landscape, Tianjin Agricultural Univercity, Tianjin 301799, China

**Keywords:** apple replant disease orchard, leaching liquor, root exudation, phenolic acids, *M. hupehensis* Rehd.

## Abstract

Organic acids secreted by plants, such as p-hydroxybenzoic acid, ferulic acid, cinnamic acid, and benzoic acid, can inhibit seed germination and root growth. The effects of root and soil leaching liquor from orchards on the growth of *M. hupehensis* Rehd. seedlings under sand culture are studied; the seedlings are sampled at 15, 30, 45, and 60 d. Changes in the amount of root exudates are determined using HPLC. Low concentrations of root leaching liquor (A1) and soil leaching liquor (B1) significantly promoted plant growth and chlorophyll synthesis; high concentrations of root leaching liquor (A6) and soil leaching liquor (B4–6) inhibited growth. Low concentrations of soil leaching liquor had no significant effect on the POD, SOD, and CAT activities. A5–6 and B5–6 significantly decreased *Fv*/*Fm* and qP values, respectively, and increased NPQ values. All root and soil leaching liquor treatments inhibited the secretion of gallic acid, hydroxybenzoic acid, benzoic acid, and phloridzin, and promoted the secretion of caffeic acid. The root leaching liquor treatments inhibited the secretion of catechin and promoted the secretion of phloretin. The soil leaching liquor treatments promoted the secretion of cinnamic acid. The secretion of other phenolic acids is likely associated with the different concentrations of leaching liquor.

## 1. Introduction

Replant disease refers to the phenomenon in which the same crop or closely related crops are repeatedly planted in the same soil and, even under normal cultivation and management conditions, their growth potential is weakened, their vulnerability to pests and diseases is aggravated, and yield and quality are reduced [1,2,3,4]. Apple (*Malus domestica*) replant disease occurs when old trees are removed from an orchard and the replanted young apple trees are short, tree vigor is weak, and their vulnerability to diseases and insect pests is aggravated, which seriously affects the yield and quality of fruit and impedes the sustainable development of the apple industry in China [5]. Its causes are manifold and include imbalances in the rhizosphere micro-ecosystem [6], the accumulation of phenolic acids [7], and the deterioration of soil physical and chemical properties [8]. Overcoming the challenges of apple replant disease is critically important for ensuring the sustainable development of the apple industry. China is the largest apple producer and consumer in the world [9]. The research on the effect of replant diseases on the environment and crops is important for minimizing losses caused by replant diseases and has major economic implications for China and all countries worldwide.

The importance of plant root secretions has been long recognized [10]. Macromolecular mucilaginous substances in root exudates include polysaccharides, phenolic compounds, and polygalacturonic acid [11]. Plant root exudates contain bioactive substances that inhibit or promote the growth of other adjacent plants or themselves, and these are referred to as allelochemicals. Apple roots secrete a large number of metabolites into the soil during growth and development, and the accumulation and transformation of these metabolites over long periods can cause disservices to trees. Toxins produced by apple roots include rhizomatites, rhizotin, light cinnamic acid, and light benzoic acid, which are associated with apple reimplantation disorders [12]. The effects of phenolic acids on plant growth mainly include the inhibition of seed germination, seedling root growth, seedling ion absorption, the activity of protective enzymes, hormone metabolism, the destruction of the structure of root cell membranes, and interference with DNA replication and RNA transcription, and other physiological and biochemical processes [13,14].

Previous studies have found that acids enter the plant through the cell membrane and alter the activity and function of some enzymes. Chlorogenic acid, caffeic acid, and catechol inhibit the activity of phosphorylase [15]. Its derivatives inhibit the hydrolytic activity of ATPase; tannic acid inhibits the activity of peroxidase (POD), catalase (CAT), and cellulase. Phenolic acids also affect the activity of phenylalanine ammonia-lyase. Callaway et al. (2000) reported that 50 μmol/L cinnamic acid and 4-hydroxy-3-methoxycinnamic acid reduce protein synthesis in lettuce seedlings [16]; 10–30 μmol/L caffeic acid, coumaric acid, ferulic acid, cinnamic acid, and vanillic acid inhibit the photosynthesis and chlorophyll content of soybean, which inhibits growth [17].

Various methods are used to collect root exudates, including the solution culture collection method and water extraction culture medium (vermiculite culture, sand culture, agar culture, and soil culture) collection method according to the culture system of the plant; root exudates can also be collected from the root system or soil [18]. Here, we conduct an experiment in which 20-year-old apple orchard continuous cropping soil and root extracts from replanted orchards are applied at different concentrations to *M. hupehensis* Rehd. seedlings under sand culture and irrigated. The growth of the seedlings is measured at 15, 30, 45, and 60 d. Changes in the content of root exudates, including the main organic phenolic acids, are analyzed. We examine the relationships of root and soil extract concentrations with various physiological indicators of seedlings at different stages, and the effects of different concentrations of soil and root extracts on the content of organic phenolic acids secreted by roots to provide insights that could aid the ability of farmers to overcome the challenges of replanting fruit trees.

## 2. Results

### 2.1. Standard Sample Chromatogram

See Figure S3 for a chromatogram of the standard PAH samples and Figure S4 for a chromatogram of the phenolic acid standard samples.

### 2.2. Effect of Soil and Root Extracts on the Growth of Seedlings of M. hupehensis Rehd.

#### 2.2.1. Effect of Root Extract on Seedling Growth

**Figure 1:**
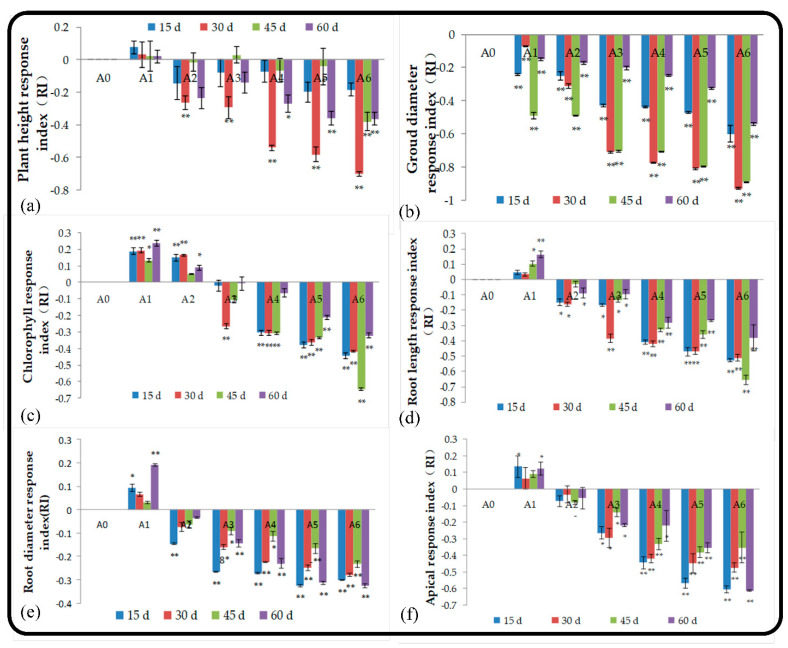
Effect of root extract on the height (**a**), diameter (**b**), and chlorophyll content (**c**), and the effect of root leaching liquor on the root length (**d**), root diameter (**e**), and number of root tips (**f**). Note: The displayed data are presented as mean ± SE (standard error) (*n* = 3). Error bars represent the standard errors of the means (*n* = 3), * correlation is significant at the 0.05 level, ** correlation is significant at the 0.01 level by Duncan’s new multiple-range test.

Figure 1a–c shows the growth status of the aboveground parts of seedlings. Lower concentrations (A1) of root extracts promoted the growth of seedlings (RI > 0), and higher concentrations resulted in stronger inhibition (RI < 0). The allelopathy of root extracts was the strongest at 30 d of seedling growth, especially for treatment A6, and the absolute value of RI reached 0.70. Any concentration of root extracts can significantly inhibit the growth of the ground diameter of seedlings The degree of inhibition increased as the concentration applied increased, and the RI value peaked on the 30th day and then decreased with time. A1 and A2 stimulated chlorophyll synthesis in the leaves, and lower concentrations were associated with stronger stimulation of chlorophyll synthesis. The inhibition of chlorophyll synthesis was highest in A6, and the average RI absolute value reached 0.46 within 60 d.

The effects of root and soil extracts on seedling roots significantly varied among treatments (Figure 1d–f). The low-concentration root extract treatment (A1) can promote root elongation and thickening and increase the number of root tips; the average RI within 60 d was 0.087, 0.095, and 0.103. When the treatment concentration was higher than that in A1, the root extract had allelopathic effects and significantly inhibited the growth of seedling roots. At 15 d, the allelopathic effects of root extracts on the root growth of seedlings were strongest, and this occurred earlier than when the strength of the allelopathic effects on the shoots was highest.

#### 2.2.2. Effect of Soil Extract on Seedling Growth

**Figure 2:**
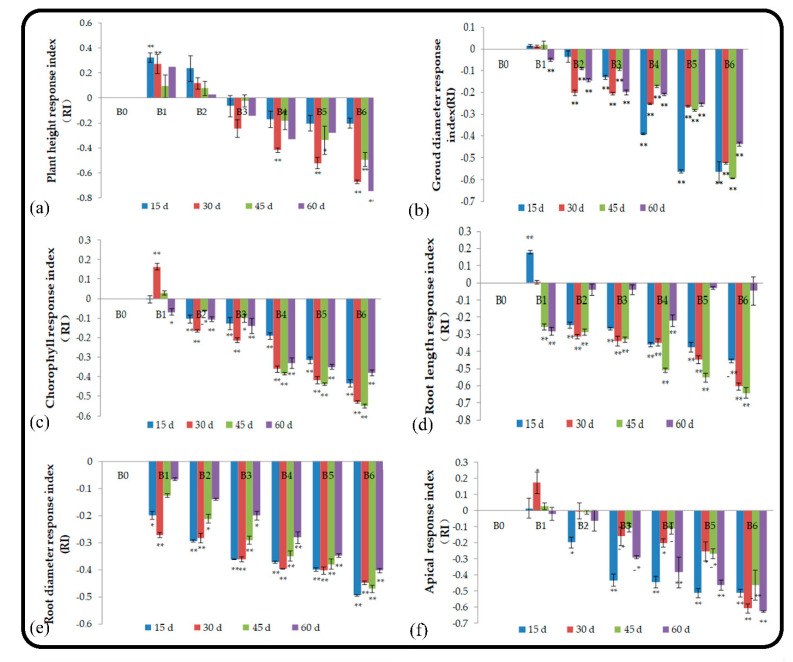
Effect of soil leaching liquor on plant height (**a**), diameter (**b**), and the chlorophyll content (**c**), and on root length (**d**), root diameter (**e**), and number of root tips (**f**). Note: The displayed data are presented as mean ± SE (standard error) (*n* = 3). Error bars represent the standard errors of the means (*n* = 3), * correlation is significant at the 0.05 level, ** correlation is significant at the 0.01 level by Duncan’s new multiple-range test.

The growth status of the aboveground parts is shown in Figure 2a–c. Seedlings treated with concentrations lower than that in B3 showed the same growth pattern as the control during the test period. Low concentrations of soil extract promoted the growth of seedlings, and growth was increased at lower concentrations. The average RI of B1 at 60 d was 0.234. After 2 months, B1 significantly promoted the growth of seedlings, and B6 significantly inhibited the growth of seedlings. The growth rate in the ground diameter of all treatments gradually increased, but was lower in all treatments compared with the control. After 45 d, all treatments had a significant inhibitory effect on the growth in ground diameter, and the absolute value of B6RI reached 0.438. When the treatment concentration was that in B1, the content of synthesized chlorophyll in the leaves was higher than that in the control in the first 30 d; it then remained the same, decreased significantly, and was lower than that of the control at 60 d. At 30 d, the chlorophyll content decreased in the other treatments with the exception of B1, and the decrease was significant in B4, B5, and B6. The low concentration of soil extract had no effect on chlorophyll synthesis over short periods, but the strength of inhibition increased as the concentration increased and the treatment time extended.

The effects of different soil extract treatments on seedling roots significantly varied (Figure 2d–f). In the first 30 d, the root elongation of *M. hupehensis* Rehd. seedlings was significantly inhibited by all treatments except treatment B1. After 2 months, treatments B1, B2, and B3 significantly inhibited root elongation, and treatments B4, B5, and B6 significantly inhibited root elongation. All treatments inhibited increases in root diameter during the experimental period, and the degree of inhibition increased with the treatment time and the concentration of leaching liquor applied. The inhibition rates of treatment B6 were 49.5%, 44.8%, 47.1%, and 47.7% at 15, 30, 45, and 60 d, respectively, and the inhibition was extremely significant. B1 could stimulate the formation of root tips in the first 45 d, which was beneficial to the absorption of nutrients by roots, and its stimulatory effect was strongest at 30 d when the number of root tips was 20.8% higher compared with that in the control; the stimulatory effect weakened, and no significant effect of leaching liquor on the formation of root tips was observed after 2 months. All the other treatments inhibited the formation of root tips, and the degree of inhibition increased as the concentration of leaching liquor applied increased. The maximum level of inhibition was observed in treatment B6 at 30 d, when the inhibition rate was 60.9% and the RI value was 0.627, indicating that allelopathy was strong.

### 2.3. Effect of Soil and Root Extracts on the Activity of Root Protective Enzymes

#### 2.3.1. Effect on POD

**Figure 3:**
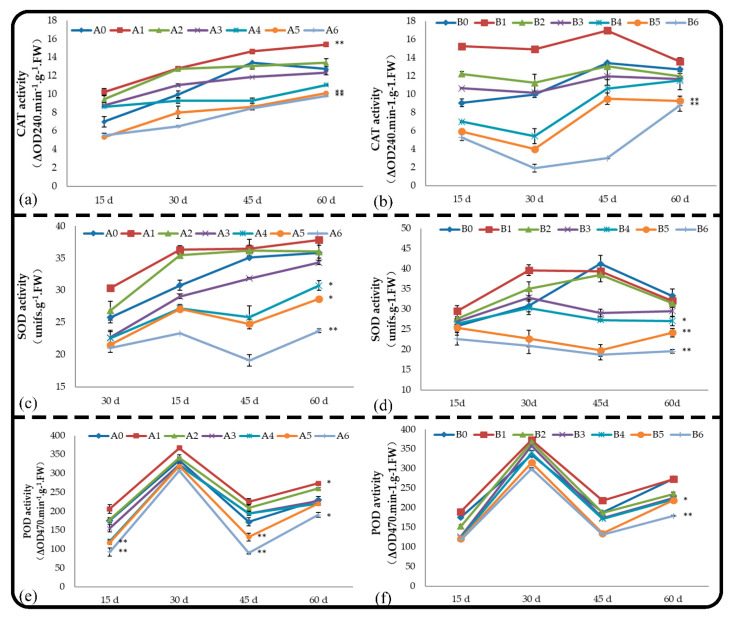
Effect of root and soil leaching liquor on POD activity (**a**,**b**), SOD activity (**c**,**d**), and CAT activity (**e**,**f**). Note: The displayed data are presented as mean ± SE (standard error) (*n* = 3). * Correlation is significant at the 0.05 level, ** correlation is significant at the 0.01 level by Duncan’s new multiple-range test.

POD mainly catalyzes the decomposition of hydrogen peroxide and some phenolic substances. The high activity of POD indicates the oxidation of membrane lipids and increases in membrane permeability, which negatively affects plant growth. Within 60 d, the root POD activity of *M. hupehensis* Rehd. seedlings treated with root extracts first increased, decreased, and then increased (Figure 3a). The activity of POD in the roots of seedlings treated with A1 and A2 was higher than that of the control, indicating that the low-concentration root extract treatment could stimulate the synthesis of POD enzymes and protect root growth. As the amount of root extract applied increased, the degree of inhibition of seedling root growth increased. The activity of POD in treatment A6 at 15 d, 30, 45, and 60 d was 52.6%, 61.9%, 52.2%, and 61.1% for the control, respectively, and the degree of inhibition was significant. The activity of POD in the roots of seedlings treated with soil and root extracts exhibited the same pattern over the 2-month period. Treatment B1 promoted the activity of POD in the first 45 d, but the effect was not significant; treatment B5 significantly inhibited the activity of POD, and treatment B6 significantly inhibited the activity of POD, indicating that the soil extract had a greater impact on the activity of POD in the roots of seedlings than the root extract.

#### 2.3.2. Effect on SOD

Many studies have shown that SOD activity increases under moderate stress levels to mediate adaptation to stress. The SOD activity of the root system of *M. hupehensis* Rehd. in the low-concentration root extract treatment and the control treatment increased with treatment time, and the SOD activity of the root system of *M. hupehensis* Rehd. in the high-concentration root extract treatment decreased at 45 d (Figure 3c). Treatments A1 and A2 increased the activity of SOD in the roots in the first 45 d, and treatments A4, A5, and A6 significantly decreased the activity of SOD in the roots by 26.5%, 29.4%, and 45.6%, respectively, at 45 d. B1, B2, and B3 promoted increases in SOD activity in the first 30 d, but as the stress treatment time extended, SOD activity decreased. Treatments B5 and B6 significantly inhibited the SOD activity of roots from the beginning of the experiment, and the degree of inhibition increased with the treatment time (Figure 3d).

#### 2.3.3. Effect on CAT

In the process of scavenging superoxide anion free radicals, SOD forms H_2_O_2_, which is harmful to cells. CAT has the function of scavenging H_2_O_2_. CAT, SOD, and POD protect the membrane system from the harm of free radicals. The CAT activity of seedling roots treated with different root extracts increased with treatment time, and this same pattern was observed in the control (Figure 3e). At 15 d, treatments with concentrations lower than A4 promoted the growth of seedlings. At 30 d, treatments with concentrations lower than A3 also promoted seedling growth. At 45 d, the CAT activity of treatment A1 was higher than that of the control. At 60 d, treatment A1 significantly promoted the activity of CAT, whereas treatments A5 and A6 significantly inhibited CAT activity, which affected seedling growth. Treatment B1 significantly promoted CAT activity in the first 45 d, but this stimulatory effect was not observed after 45 d. Treatments B5 and B6 significantly inhibited the activity of CAT in the roots of *M. hupehensis* Rehd. seedlings. The low-concentration soil extract treatments promoted seedling growth and enzyme activities; however, as the treatment time and concentration of leaching liquor applied extended, the degree of stress increased, the strength of allelopathy increased, and the seedlings finally lost their resistance, which led to decreases in root protective enzyme activities.

### 2.4. Effect of Soil and Root Extracts on the Chlorophyll Fluorescence of M. hupehensis Rehd. Seedlings

Pn only reflects the apparent photosynthetic capacity, and the specific internal mechanism affecting photosynthesis can be revealed by chlorophyll fluorescence parameters. The chlorophyll fluorescence parameters of *M. hupehensis* Rehd. seedlings in different treatments were measured, and the maximum photochemical efficiency of PSII was measured using the following formula: *F_v_*/*F_m_* = (*F_m_* − *_m_*/*F_m_*). The photochemical quenching coefficient was measured using the following formula: qP = (*F_m_*′ − *F_s_*)/(*F_m_*′ − _m_′). The non-photochemical quenching coefficient was measured using the following formula: NPQ = (*F_m_* − *F_m_*′)/*F_m_*′.

#### 2.4.1. Effect on *F_v_/F_m_*

**Figure 4:**
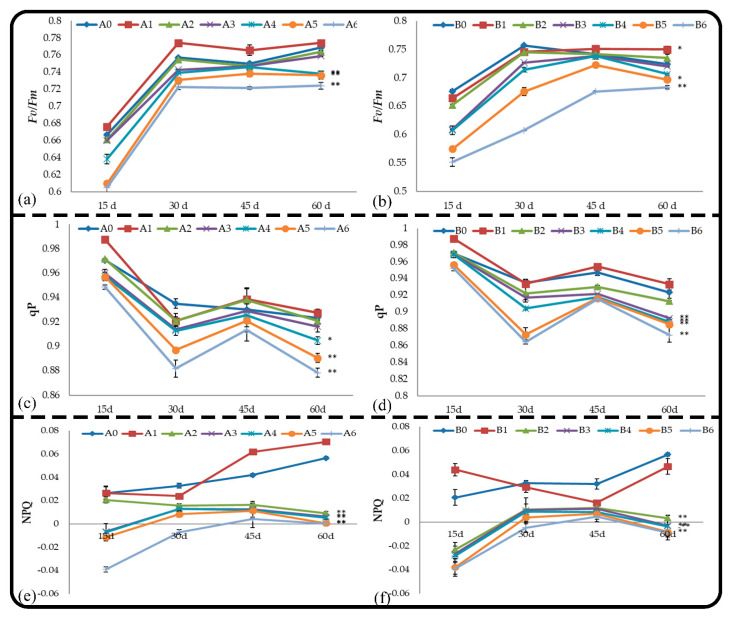
Effect of root and soil leaching liquor on *Fv*/*Fm* (**a**,**b**), qP (**c**,**d**), and NPQ (**e**,**f**). Note: The displayed data are presented as mean ± SE (standard error) (*n* = 3). * Correlation is significant at the 0.05 level, ** correlation is significant at the 0.01 level by Duncan’s new multiple-range test.

*F_v_/F_m_* represents the primary light-energy-conversion efficiency and the potential quantum efficiency of PSII, which is also known as the maximum photochemical efficiency of PSII and is proportional to the photosynthetic electron-transport activity. The effects of different concentrations of root and soil extracts on *F_v_/F_m_* within 60 d were consistent in the treatments and the control (Figure 4). The *F_v_/F_m_* values of the seedlings treated with low concentrations of leaching liquor were higher than those of the control (Figure 4a), but no significant difference was observed between treatments and the control. Treatments A5, A6, and A7 had significant inhibitory effects on *F_v_/F_m_* throughout the experiment. The *F_v_/F_m_* of seedlings treated with high concentrations of soil extract significantly decreased at the beginning of the treatment compared with the control, and the difference was most significant at 30 d; although this difference subsequently decreased, it remained significant.

#### 2.4.2. Effect on qP

The photochemical quenching coefficient (qP) is the proportion of light energy absorbed by PSII antenna pigments for photochemical reaction electron transfer, that is, the proportion of excitation energy entering the photosynthetic electron-transport system through QA oxidation for photochemical assimilation. It is also a measure of the oxidation state of the primary electron acceptor QA, which reflects the relative number of PSII open centers. Larger qP values indicate a greater amount of QA- is reoxidized to QA and a higher probability of PSII electron transfer. Within 2 months, the effects of root and soil extract treatments on the leaf qP of *M. hupehensis* Rehd. seedlings were similar (Figure 4c,d); leaf qP first decreased, increased, and then decreased. Treatments A5, A6, B3, B4, B5, and B6 significantly inhibited leaf qP.

#### 2.4.3. Effect on NPQ

The non-photochemical quenching coefficient NPQ reflects the light energy absorbed by PSII antenna pigments that cannot be used for photosynthetic electron transport and dissipates in the form of heat. Patterns of change in NPQ were opposite that of qP. The effects of root and soil extracts on NPQ were consistent. Low = concentration leaching liquor treatments had no effect on the NPQ value; the high-concentration treatments A5, A6, B5, and B6 significantly increased the NPQ value (Figure 4e,f).

### 2.5. Effect of Soil and Root Extracts on the Phenolic Acids Secreted from the Roots of M. hupehensis Rehd. Seedlings

#### 2.5.1. Catechin

**Figure 5:**
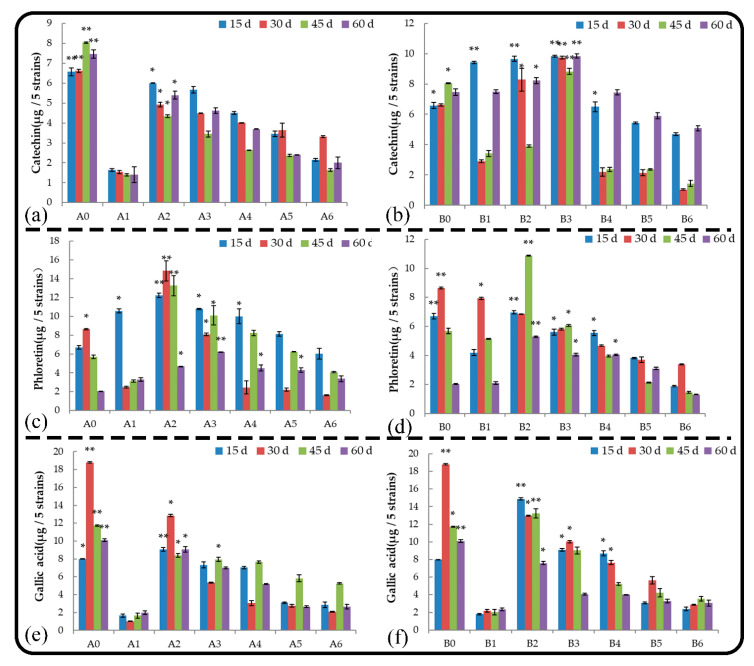
Effect of root and soil leaching liquor on the secretion of catechin (**a**,**b**), phloretin (**c**,**d**), and gallic acid (**e**,**f**). Note: The displayed data are presented as mean ± SE (standard error) (*n* = 3). * Correlation is significant at the 0.05 level, ** correlation is significant at the 0.01 level by Duncan’s new multiple-range test.

Catechins and their oxidation products are ternary compounds of carbon, hydrogen, and oxygen that are synthesized from sugars through a series of enzymes that mediate the formation of benzene-ring compounds through the shikimic acid pathway. Catechins are phenolic substances that can precipitate heavy metals and proteins [19]. Under normal growth conditions, catechin is the main substance secreted by the root system of *M. hupehensis* Rehd. seedlings in the late growth stage, and treatments B3, B4, B5, and B6 had no effect on catechin secretion; however, the other treatments promoted catechin secretion of the root system, as catechin was the main component secreted in the early growth stage of the root system (Figure 5a,b). Treatments B5 and B6 significantly inhibited the secretion of catechins in the roots of *M. hupehensis* Rehd. seedlings, and higher concentrations of leaching liquor resulted in a stronger inhibition of catechin secretion. There were significant differences in the effects of root and soil extracts applied at the same concentration on the catechin secretion of seedlings, and the amount of catechin secreted by the roots of seedlings treated with soil extracts was higher than that secreted by the roots of seedlings treated with root extracts.

#### 2.5.2. Phloretin

Phloretin mainly exists in the peel and root bark of juicy fruits, such as apples and pears. Under normal growth conditions, phloretin was the main substance secreted by the roots of *M. hupehensis* Rehd. seedlings during the early stage of growth (Figure 5c,d). Treatments B2 and B3 delayed the secretion of phloretin, and the other treatments had no effect on phloretin secretion. Root extracts played a major role in promoting the secretion of phloretin in the roots of *M. hupehensis* Rehd. seedlings. At the end of the treatment, the stimulatory effect first increased and then decreased as the concentration applied increased, and the stimulatory effect was extremely significant. At the end of the treatment period, B1 had no effect on the secretion of phloretin, B6 significantly inhibited the secretion of phloretin, and the other treatments had significant stimulatory effects; the strength of the stimulatory effect decreased as the concentration of leaching liquor applied increased. The effects of root extract on phloretin secretion were stronger than the effects of soil extract when the same concentration of leaching liquor was applied.

#### 2.5.3. Gallic Acid

Gallic acid is an important secondary metabolite of plants, and its secretion is affected by both genetic factors and the environment. Under normal growth conditions, gallic acid is the main substance secreted by the roots of *M. hupehensis* Rehd. seedlings during the early stage of growth (Figure 5e,f). Root extract treatment delayed the secretion of gallic acid by the roots, whereas soil extract treatment had no effect on gallic acid secretion. Root and soil extract treatments mainly inhibited the secretion of gallic acid by the roots. At 15 d, treatments A2, B2, B3, and B4 promoted the secretion of gallic acid from *M. hupehensis* Rehd. roots for a short period, and the secretion of gallic acid was inhibited as the treatment time extended. The exudation of gallic acid was significantly inhibited by treatments A1 and B1 and high concentrations of root and soil extracts; high concentrations of root and soil extracts resulted in strong inhibition. When the concentration of the extracts was higher than that of A4 and B4, there was no significant difference in the degree of inhibition of gallic acid secretion among treatments.

#### 2.5.4. Chlorogenic Acid

**Figure 6:**
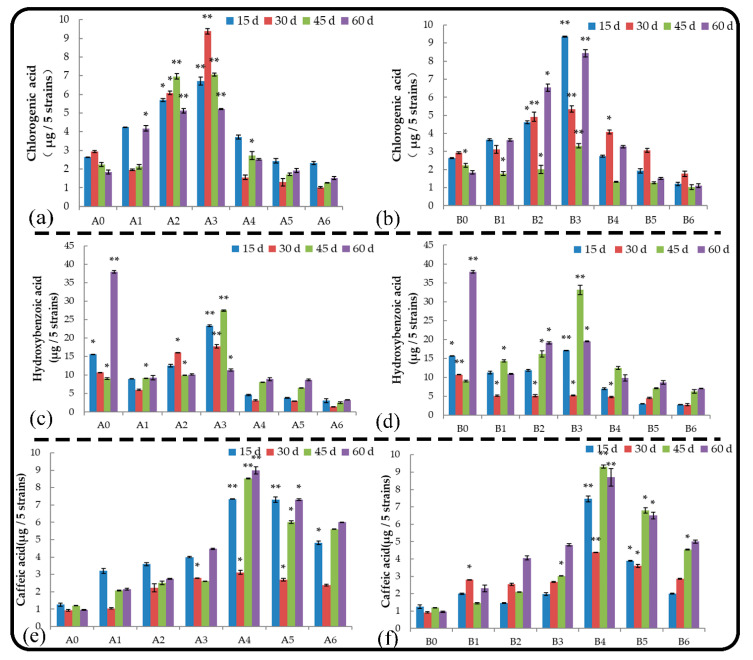
Effect of root and soil leaching liquor on the secretion of chlorogenic acid (**a**,**b**), hydroxybenzoic acid (**c**,**d**), and caffeic acid (**e**,**f**). Note: The displayed data are presented as mean ± SE (standard error) (*n* = 3). * Correlation is significant at the 0.05 level, ** correlation is significant at the 0.01 level by Duncan’s new multiple-range test.

Chlorogenic acid is a depside formed by caffeic acid and quinic acid; it is a phenylpropanoid compound produced by the shikimate pathway during the aerobic respiration of plants. Under normal growth conditions, chlorogenic acid was the main substance secreted by the root system of *M. hupehensis* Rehd. seedlings during the early stage of growth (Figure 6a,b). Treatments A2 and B2 delayed the secretion of chlorogenic acid by the root system, and other treatments had no effect on chlorogenic acid secretion. Within 2 months, treatments A1, A2, and B3 significantly promoted the secretion of chlorogenic acid by roots, and the stimulatory effect first increased and then decreased with time; treatments A5, A6, and B6 significantly inhibited the secretion of chlorogenic acid by the roots. The amount of chlorogenic acid secreted by the roots of *M. hupehensis* Rehd. seedlings under each root extract treatment was higher than that under each soil extract treatment in the same low-concentration treatments, and the amount of chlorogenic acid secreted was lower in these treatments compared with the high-concentration soil extract treatments.

#### 2.5.5. Para-Hydroxybenzoic Acid

Hydroxybenzoic acid is one of the main root exudates of *M. hupehensis* Rehd; it is also one of the main autotoxic substances secreted by strawberry roots, and it has a significant inhibitory effect on the growth of roots. Under normal growth conditions, p-hydroxybenzoic acid is the main substance secreted by the root system of *M. hupehensis* Rehd. seedlings during the late growth stage (Figure 6c,d), and no treatments affected p-hydroxybenzoic acid secretion. Treatment A3 significantly promoted the secretion of p-hydroxybenzoic acid in the first 45 d and then strongly inhibited its secretion. Within 2 months, root extracts of treatments A5 and A6 and soil extracts of all treatments (except treatment B3) significantly inhibited the secretion of p-hydroxybenzoic acid by the roots of seedlings, and the inhibitory effect of root extracts was stronger than that of soil extracts when they were applied at the same concentrations.

#### 2.5.6. Caffeic Acid

Caffeic acid is one of the main allelochemicals secreted by plant roots, and it is mainly produced by the secondary metabolism of shikimic acid. Under normal growth conditions, caffeic acid is the main substance secreted by the roots of *M. hupehensis* Rehd. seedlings during the early growth stage, and treatments A1, A2, and B1 had no effect on caffeic acid secretion (Figure 6e,f); caffeic acid secretion by the roots was delayed in the other extract treatments. Root extracts and high concentrations of soil extracts promoted the secretion of caffeic acid from the roots of *M. hupehensis* Rehd. seedlings, and the magnitude of the stimulatory effect first increased and then decreased as the amount of leaching liquor applied increased.

#### 2.5.7. Vanillin

**Figure 7:**
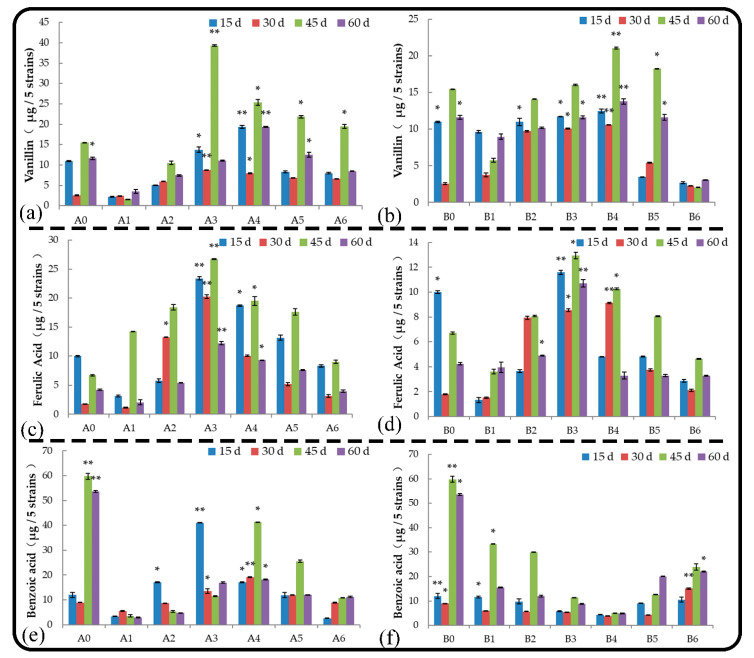
Effect of root and soil leaching liquor on the secretion of vanillin (**a**,**b**), ferulic acid (**c**,**d**), and benzoic acid (**e**,**f**). Note: The displayed data are presented as mean ± SE (standard error) (*n* = 3). * Correlation is significant at the 0.05 level, ** correlation is significant at the 0.01 level by Duncan’s new multiple-range test.

Vanillin is one of the main allelochemicals secreted by roots; it can inhibit the growth of plant roots, destroy the structure of cell membranes, and inhibit seed germination. Under normal growth conditions, vanillin was the main substance secreted by the root system of *M. hupehensis* Rehd. seedlings during the late growth stage (Figure 7a,b). Treatment B1 promoted vanillin secretion by the root system, and other treatments had no effect on vanillin secretion. Within 2 months, treatments A4 and B4 significantly promoted vanillin secretion from the roots, and treatments A1 and B1 significantly inhibited vanillin secretion from the roots. Vanillin secretion was significantly lower when roots were treated with the same low concentrations of root extract compared with soil extract, and the opposite effect was observed when high concentrations of extracts were applied.

#### 2.5.8. Ferulic Acid

Ferulic acid is one of the derivatives of cinnamic acid. Ferulic acid was first detected in the seeds and leaves of plants. It is a phenolic acid that is widespread in plants. It combines with polysaccharides and proteins in the cell wall to form the skeleton of the cell wall. It is also one of the main allelochemicals secreted by roots. Root and soil extract treatments delayed the secretion of ferulic acid by the roots (Figure 7c,d). The effects of root and soil extracts on the secretion of ferulic acid from *M. hupehensis* Rehd. seedlings were consistent as the amount of leaching liquor applied increased. A stimulatory effect of leaching liquor on ferulic acid secretion was observed as the amount of leaching liquor applied increased; however, beyond a certain value, the stimulatory effect decreased, and ferulic acid secretion was observed. Treatments A3, A4, and A5 significantly promoted the secretion of ferulic acid in the roots, and treatment A3 had the most significant effect. The secretion of ferulic acid by the roots was three times higher in treatment A3 compared with the control. The effect of soil extract on the secretion of ferulic acid by the roots was relatively weak, and the effect of treatment B3 was significant compared with that of the control. The amount of ferulic acid secreted by the roots was 0.6 times higher in treatment B3 compared with the control.

#### 2.5.9. Benzoic Acid

Benzoic acid is one of the main root exudates of *M. hupehensis* Rehd. It has significant allelopathic effects, destroys membrane permeability, causes root cell membrane dysfunction, and affects the absorption of nutrients by roots. Under normal growth conditions, benzoic acid was the main substance secreted by the root system of *M. hupehensis* Rehd. seedlings during the late growth stage (Figure 7e,f). Low-concentration root extract treatments advanced benzoic acid secretion, and the other treatments had no effect on benzoic acid secretion. In the later stage of the treatments, the root and soil extracts mainly inhibited the secretion of benzoic acid by seedlings, and the inhibition was strongest in treatments A1, A2, B3, and B4. The inhibition rates of treatments A1 and B4 were 74.75% and 75.66%, respectively, and there was no significant difference between them. In the root extract treatments, the amount of benzoic acid secreted by roots first increased and then decreased as the amount of root extract applied increased. In the soil extract treatments, the amount of benzoic acid secreted first decreased and then increased as the amount of soil extract applied increased, and this pattern was opposite the effect of root extracts. Benzoic acid secretion was significantly higher in A3 and A4 than in B3 and B4, respectively, and benzoic acid secretion was significantly lower in the root extract treatments than in the soil extract treatments at other concentrations.

#### 2.5.10. Phloridzin

**Figure 8:**
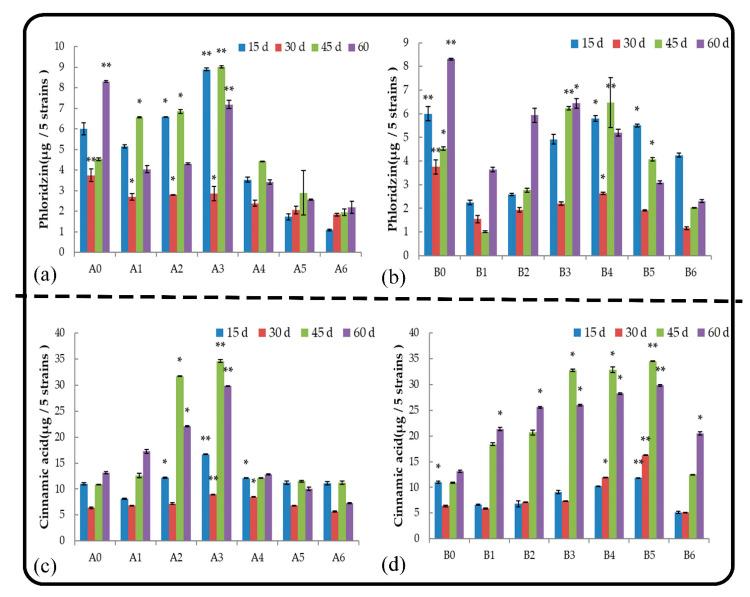
Effect of root and soil leaching liquor on the secretion of phloridzin (**a**,**b**) and cinnamic acid (**c**,**d**). Note: The displayed data are presented as mean ± SE (standard error) (*n* = 3). * Correlation is significant at the 0.05 level, ** correlation is significant at the 0.01 level by Duncan’s new multiple-range test.

Phloridzin is a glycoside that is abundant in the bark, root system, and other organs of apple trees. A large amount of phloridzin is released into the soil via root secretion and residue decomposition, and this results in a decline in apple tree growth, yield, and quality. Phloridzin was the main substance secreted by the root system of *M. hupehensis* Rehd. seedlings during the late growth stage under normal growth conditions (Figure 8a,b), and all treatments had no effect on phloridzin secretion. The amount of phlorizin secreted first increased and then decreased as the concentration of leaching liquor applied increased. Root and soil extract treatments inhibited phloridzin secretion by seedlings. Phloridzin secretion by seedlings was higher when they were treated with root extract than with soil extract at the same low concentrations; the opposite pattern was observed in the high-concentration treatments.

#### 2.5.11. Cinnamic Acid

Cinnamic acid, which is produced by the deamination of phenylalanine, is the main root exudate of *M. hupehensis* Rehd. It affects plant growth by affecting ion absorption, water absorption, photosynthesis, and protein and DNA syntheses. Low-concentration root extract treatments and all soil extract treatments promoted the secretion of cinnamic acid by the roots (Figure 8c,d), and the stimulatory effect first increased and then decreased as the concentration of extract applied increased. At 45 d, A3 had the strongest stimulatory effect among root extract treatments, and cinnamic acid secretion was 2.17 times higher in A3 compared with that of the control. B5 had the strongest stimulatory effect in the treatment of soil extract, and cinnamic acid secretion was 2.15 times higher in B5 compared with that of the control. At the end of the treatments, low concentrations of root extracts promoted the secretion of cinnamic acid by the roots, and high concentrations of root extracts had a strong stimulatory effect on cinnamic acid secretion. All soil extract treatments significantly promoted the secretion of cinnamic acid. The stimulatory effect first increased and then decreased as the concentration applied increased. The cinnamic acid secretion of seedlings treated with soil extract was higher than that treated with root extract.

#### 2.5.12. Correlation between the Growth of *M. hupehensis* Rehd. and Catechin, Phloretin, and Gallic Acid Secretions

**Figure 9:**
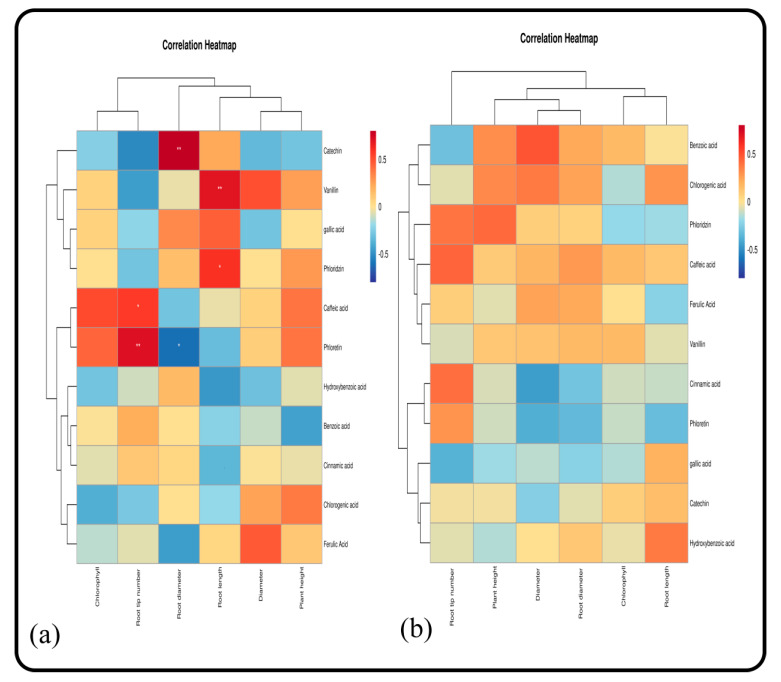
Correlations between seedling growth and phenolic acids of *M. hupehensis* Rehd. at different stages: 15 d (**a**) and 30 d (**b**). Note: * Correlation is significant at the 0.05 level, ** correlation is significant at the 0.01 level.

**Figure 10:**
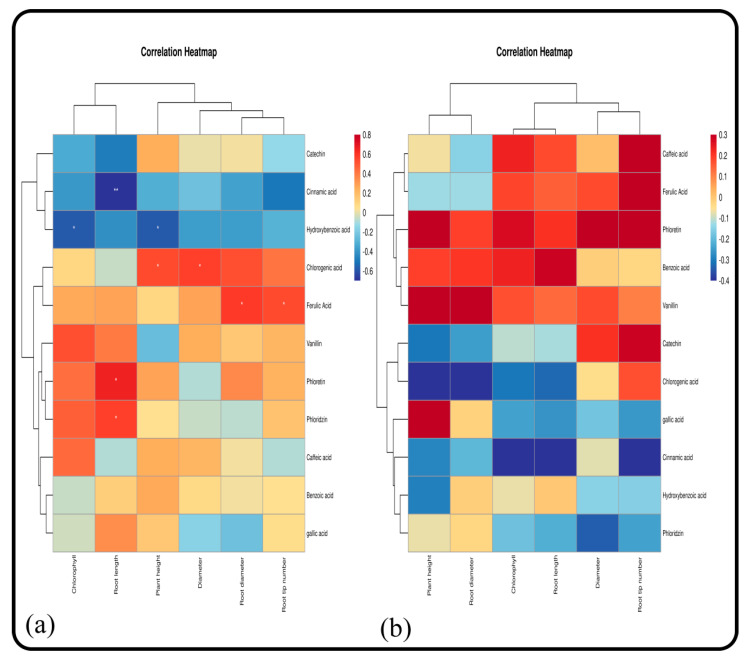
Correlations between seedling growth and the phenolic acids of *M. hupehensis* Rehd. at different stages: 45 d (**a**) and 60 d (**b**). Note: * Correlation is significant at the 0.05 level, ** correlation is significant at the 0.01 level.

Catechin, phloretin, and gallic acid are the main components of root exudates of *M. hupehensis* Rehd. seedlings. In this study, the correlations between the growth of *M. hupehensis* Rehd. seedlings and the amount of catechin, phloretin, and gallic acid secreted by roots was analyzed. Under normal growth conditions, the secretion of catechin was not related to plant height, ground diameter, and the chlorophyll content (Figure 9 and Figure 10). There was a significant positive correlation in the secretion of these three root exudates between the root and soil extract treatments. Phloretin secretion was significantly correlated with plant height and basal diameter, and the correlation disappeared after treatment with root and soil extracts. The secretion of gallic acid was not correlated with plant height, basal diameter, and the chlorophyll content, but it was significantly positively correlated with plant height and chlorophyll after treatment with root extract and significantly positively correlated with seedling growth after treatment with soil extract.

## 3. Discussion

Phenolic acids, including chlorogenic acids, are major polyphenolic compounds in Jerusalem artichoke (*Helianthus tuberosus* L.). Phenolic acids are common allelochemicals in soil [20,21,22]. Our findings indicated that low concentrations of root and soil extracts could promote the growth of *M. hupehensis* Rehd. seedlings, and the degree of growth inhibition was positively correlated with the amount of extract applied. Growth inhibition first increased, decreased, and then increased as the treatment time extended. Allelopathy was positively correlated with the treatment concentration, and the strength of allelopathy first increased and then decreased as the treatment time extended. Low-concentration soil extract treatments had no significant effect on the ground diameter of seedlings and the synthesis of chlorophyll in leaves. The high-concentration root and soil extract treatments inhibited increases in ground diameter and the synthesis of porphyrin, accelerated the decomposition of chlorophyll, reduced the content of chlorophyll in leaves, and caused the leaves lose their green appearance [23]. The degree of inhibition was positively correlated with the concentration of leaching liquor applied. Changes in seedling height, net growth rate of ground diameter, and chlorophyll content were analyzed, and the seedling height and net growth rate of ground diameter were higher in the soil extract treatments than in the root extract treatments when the same concentrations of extract were applied. The content of phenolic acid allelochemicals in the root extract was higher than that in the soil extract, and phenolic acid and other allelochemicals have been shown to have significant effects on the aboveground growth of crops [24]. The chlorophyll content was lower in the soil extract treatments than in the root extract treatments when the same concentration of extract was applied.

Changes in the chlorophyll fluorescence of the leaves of *M. hupehensis* Rehd. seedlings in the different root and soil extract treatments were studied. When low concentrations of these extracts were applied, they increased the chlorophyll fluorescence of the leaves. However, the extracts did not have a stimulatory effect on the chlorophyll fluorescence when they were applied at high concentrations. The mechanism of the photosynthetic system was disrupted, the rate of photosynthetic electron transport decreased, the number of PSII open centers decreased, the proportion of light energy consumed in the form of heat dissipation increased, photosynthesis was hindered, and the growth of seedlings was inhibited.

The pollution of orchard soil is becoming increasingly widespread. There are several organic pollutants in soil aside from phenolic acid allelochemicals. We detected the content of PAHs in the soil, and the level of PAH pollution in the orchard soil was moderate (833.88 ng/G); the high ecological risks associated with PAH pollution can have adverse effects on crops. Ren et al. pointed out that allelochemicals can significantly inhibit the growth of cucumber roots [25]. The results of this study show that low concentrations of root extract can promote root elongation and thickening and stimulate root tip differentiation. However, above a certain concentration, root extracts can inhibit root growth and have allelopathic effects. Replant stress would thus have an adverse effect on the growth of crop roots, and this is consistent with the results of previous studies. During the experimental period, the root elongation of *M. hupehensis* Rehd. seedlings was significantly inhibited by the soil extract treatment, and the degree of inhibition was positively correlated with the concentration applied. Arabidopsis thaliana can absorb the three-ring PAH phenanthrene, and symptoms of phenanthrene stress, including root and seedling growth inhibition, fragrant hair tuft deformities, and root hair reductions, were observed [26]. Phenanthrene, pyrene, and 1,2,4-trichlorobenzene can inhibit the root elongation of taller plants (wheat, cabbage, and tomato), and there was a significant linear or logarithmic correlation between the concentration of leaching liquor applied and the inhibition rate of root elongation (*p* = 0.05) [27]. The results of these studies are consistent with the results of this experiment and confirm that the presence of PAHs in the soil has adverse effects on crops.

The root system is the first organ of plants exposed to harmful substances, and changes in root structure directly reflect the effects of harmful substances on plant growth and development [28]. Allelopathic effects on the roots of *M. hupehensis* Rehd. seedlings treated with root extract appeared earlier and were stronger compared with the shoots of seedlings. These findings indicated that phenolic acids that initially accumulated in the roots are not transported upward but accumulate in the roots, and the roots are damaged first. When the concentration exceeded a certain level, the phenolic acids accumulated in the roots and were transported to the shoot, which resulted in poor growth of the aboveground parts. Soil extracts had effects on the shoot and root growth of *M. hupehensis* Rehd. seedlings at the beginning of the treatment period, but the effect on root growth was stronger than that on shoot growth. This might stem from the fact that the root system absorbs PAHs in the soil and transports them upward; although the root system is affected by PAHs, the growth of the aboveground parts is also inhibited. However, the amount of PAHs transported upward is less than the amount of PAHs that the root system is exposed to, and this might explain why the root system is affected to a greater degree compared with the aboveground parts. The root parameters of seedlings treated with soil extract were lower than those of seedlings treated with root extract when soil and root extracts were applied at the same concentrations, and the effect of soil extract on the growth of seedling roots was greater. A large number of microorganisms and PAHs occur in the soil in addition to phenolic acid allelochemicals, and these can have major effects on the development of the roots of seedlings and play an important role in mediating the effects of apple replant disease.

Recent studies have shown that plants produce more oxygen free radicals under stress conditions, and this aggravates the peroxidation of the plasma membrane and leads to the destruction of the membrane system [29]. The results of this study show that the allelopathic effects of root extract are closely related to the concentration of root extract applied. Changes in the POD activity of *M. hupehensis* Rehd. roots were similar among treatments: first increasing, decreasing, and then increasing. Low-concentration treatments can promote increases in POD and CAT activities, indicating that within a certain time and at certain concentrations, plants can activate their own defense mechanisms to resist stress [30]; intermediate concentrations are beneficial to the growth of plants. The activities of POD and CAT were not significantly affected by leaching liquor application. The activities of POD and CAT in roots of *M. hupehensis* Rehd. seedlings were significantly inhibited by the high-concentration treatments, the activities of enzymes were reduced, the protective mechanism in response to stress was ineffective, and the growth of crops was affected; these findings are consistent with the results of previous studies. Low-concentration treatments did not affect the growth of *M. hupehensis* Rehd. seedlings; these treatments even promoted increases in the activity of protective enzymes, root respiration, the growth of the root system, the synthesis of chlorophyll, and other physiological functions. The high-concentration treatments decreased the activity of root protective enzymes, weakened the molecular oxygen and H_2_O_2_-scavenging ability of the antioxidative defense system, and led to an imbalance in the production and elimination of active oxygen, the accumulation of oxygen free radicals, increases in the content of membrane peroxidation products, and disruption of the structure and function of the cell membrane. These effects alter the structure of chloroplasts and mitochondria and the content of hormones, and damages the ultrastructure of chloroplasts and mitochondria, which affects photosynthesis and inhibits the normal growth of plants [31,32].

The research on the phenolic acids secreted by crop roots under stress conditions has mainly focused on the effects of replant stress factors, such as heavy metals, nutrient deficiencies, and organic pollution [33]. In this experiment, the effects of root and soil extract stress on the secretion of phenolic acids from the roots of *M. hupehensis* Rehd. seedlings were studied. Eleven phenolic acids were detected in the root exudates of *M. hupehensis* Rehd. seedlings in all treatments, and the amount of phenolic acids secreted by seedlings varied among treatments. Replant stress significantly inhibited the secretion of p-hydroxybenzoic acid, benzoic acid, and phlorizin. The degree of inhibition of p-hydroxybenzoic acid secretion by the soil extract was greater than that of root extract when they were applied at the same concentrations. B3 and B4 had stronger inhibitory effects on benzoic acid than root extracts, and the opposite effect was observed for other concentrations of root extracts. The degree of inhibition of phlorizin associated with the application of low concentrations of soil extract was greater than that of the root extract, and the opposite pattern was observed when high concentrations were applied. P-hydroxybenzoic acid, benzoic acid, and phloridzin can affect plant growth by affecting cell membrane permeability, ion absorption, water absorption, photosynthesis, and protein and DNA syntheses [34,35,36]. Yin et al. showed that these three substances can significantly inhibit the growth of *M. hupehensis* Rehd. seedlings. This indicates that these three substances exist in large quantities in the root system and soil extract, which exerts feedback inhibition on root secretion [37].

Replant stress mainly promotes the secretion of caffeic acid and chlorogenic acid by the root system of *M. hupehensis* Rehd. These two phenolic acids are the main allelochemicals secreted by the root system of alfalfa [38] and can significantly inhibit the growth and development of soybean. The accumulation of caffeic acid and chlorogenic acid in soil further aggravated replant disease, had negative effects on the growth of crops, promoted the secretion of organic acids of crops, and increased the difficulty of overcoming the effects of replant disease. Vanillic acid reduces the absorption of 32P and methionine by soybean roots [39]. The inhibition of vanillin by low concentrations of soil extract was greater than that by root extract, and the opposite pattern was observed when high concentrations were applied. Treatments A1, A2, B1, and B2 promoted the growth of crop roots, significantly inhibited the secretion of vanillin by roots, reduced the accumulation of vanillin in soil, and alleviated the challenges of apple replant disease in orchards. All soil extract treatments and low-concentration root extract treatments significantly promoted the secretion of cinnamic acid. The stimulatory effect of root extracts was weaker than that of soil extracts when the same concentrations of root and soil extracts were applied. Cinnamic acid damages the cell membrane of the root epidermal cells of cucumber seedlings, affects the absorption of nutrients by roots, and inhibits the germination of crop seeds and the growth of seedlings.

Ferulic acid is a derivative of cinnamic acid. Treatments A1 and B6 promoted the secretion of cinnamic acid but inhibited the secretion of ferulic acid, indicating that these two treatments inhibited the conversion of cinnamic acid to ferulic acid.

Phloridzin lacks only one beta-D-glucopyranosyloxy group compared with Phloretin [40]. Replant stress inhibits the secretion of phloridzin by roots and promotes the secretion of phloridzin. This might stem from the fact that replant stress inhibits the addition of one beta-D-glucopyranosyloxy group to phloridzin or causes phlorizin to lose a beta-D-glucopyranose oxygen group to become phloretin; the specific conversion mechanism requires clarification. Under normal growth conditions, phloretin secretion was significantly positively correlated with plant height and significantly negatively correlated with ground diameter, indicating that phloretin affected the growth of *M. hupehensis* Rehd. seedlings; this suggests that allelochemicals secreted by the roots of *M. hupehensis* Rehd. seedlings significantly inhibited increases in ground diameter. The specific mechanism underlying this effect requires further study.

Gallic acid and catechin are the main components of the root exudates of *M. hupehensis* Rehd. Under normal growth conditions, the secretion of gallic acid and catechin was not correlated with plant height, basal diameter, and the chlorophyll content. After treatment with root and soil extracts, the secretion of gallic acid and catechin by roots was inhibited, but the secretion of these compounds was significantly positively correlated with seedling growth. Gallic acid and catechin in root exudates were allelochemicals of *M. hupehensis* Rehd. seedlings, but they did not have a negative effect on crop growth; by contrast, they might stimulate crop growth under replant disease conditions. The specific mechanism underlying this possibility requires further study.

## 4. Materials and Methods

### 4.1. Experimental Materials and Treatments

The experiment was conducted at the State Key Laboratory of Crop Biology at Shandong Agricultural University in Tai’an City, Shandong Province, China. *M. hupehensis* Rehd. (purchased from Shandong Horticultural Techniques & Services Co. Ltd., Tai’an, Shandong, China) was sterilized with 0.3% H_2_O_2_ for 20 min and then rinsed with sterile water. In January 2020, the seeds were buried in sand for 65 d. After the seeds were exposed, they were sown in sand culture in small flowerpots, with 10 seeds in each pot, and regularly irrigated with Hoagland nutrient solution. When the seedlings had 4–5 leaves, the seedlings were thinned, and 5 seedlings with relatively consistent growth were placed in each pot. When the seedlings were fully developed with 6–7 leaves, each seedling was treated with 1 mL of treatment solution. Root secretions were collected every 15 d in 3 biological replicates; root exudates were also collected on a regular basis.

### 4.2. Preparation of Root and Soil Extracts

The roots and soil were collected from a 20-year-old Fuji (Fuji/Malus × robusta (Carri CarriŠre) Rehder) replanted orchard in a suburb of Taian. The soil was brown loam, the soil bulk density was 1.31 G·cm^−3^, and the pH was 5.61. The content of soil nutrients was as follows: 4.56 mg·kg^−1^ ammonium nitrogen, 7.38 mg·kg^−1^ nitrate nitrogen, 34.82 mg·kg^−1^ available phosphorus, 62.54 mg·kg^−1^ available potassium, and 7. 92 G·kg^−1^ organic matter.

According to differences in root diameter, the root system was divided into thick roots (diameters larger than 2 cm), middle roots (diameters 1–2 cm), and thin roots (diameters smaller than 1 cm). The weight ratio used in this experiment was thick heel: middle root:fine root = 1:1:1.The root system was mixed with equal amounts of water and shaken for at least 24 h; the samples were added to stock solution and stored at a low temperature (4 °C) for subsequent use. The stock solution was diluted and used as the solution to treat seedlings (Table S1).

For the preparation of soil extract, soil samples were collected in layers (0–15 cm, 15–30, and 30–45 cm) near the root system using the 4-point method and mixed in equal proportions. After the samples were air-dried, they were sieved through a 60-mesh sieve. The soil and water were mixed and shaken for 24 h; they were then filtered and stored at a low temperature (4 °C), and the stock solution was diluted and used to treat the seedlings (Table S1).

### 4.3. Preparation of Standard Solution

Preparation of PAHs standard solution: 16 types of USEPA priority PAH standard samples were purchased from O2si Smart Solutions, and each ml of acetonitrile contained 197.6 mg of Naphthalene (Nap, 2 rings), 198.1 mg of Dihydroacenaphthene (Acy, 2 rings), 198.1 mg of Acenaphthene (Ace, 2 rings), 198.1 mg of Dihydroacenaphthene (Acy, 2 rings), 198.1 mg of Dihydroacenaphthene (Acy, 2 rings), 198.1 mg of Dihydroac (2-ring) 199.1 mg of Fluorene (Flu, 2-ring), 198.3 mg of Phenanthrene (Phe, 3-ring), 198.1 mg of Anthracene (Ant, 3-ring), 198.2 mg of Fluoranthene (Fla, 3-ring), 198.1 mg of Pyrene (Pyr, 4-ring), 203.2 mg of Chrysene (Chr, 4-ring), 197.9 mg of Benzo [a] anthracene (Baa, 4-ring), 196.8 mg of Benzo [B] fluoranthene (Bbf, 4-ring), 197.5 mg of Benzo [K] fluoranthene (Bkf, 4-ring), 197.6 mg of Benzo [a] pyrene (Bap, 4-ring), 199.4 mg of Indeno [1,2,3, -cd] pyrene (Ilp, Dibenz [a, H] anthracene (Dba, 4-ring), and 197.6 mg of Benzo [G, H, I] pyrene (Bgp, 6-ring). Structures of the 16 PAHs are shown in Figure S1.

The preparation of phenolic acid standard solution was as follows: gallic acid, chlorogenic acid, catechin, caffeic acid, vanillin, benzoic acid, phloridzin, and phloretin were purchased from German Sigma Company; p-hydroxybenzoic acid was purchased from Beijing Xizhong Chemical Plant; ferulic acid was purchased from Shanghai No.1 Reagent Factory; and cinnamic acid was purchased from Tianjin Yuanhang Chemical Co., Ltd. (Tianjin, China). Gallic acid (1.3 mg), chlorogenic acid (1.5 mg), hydroxybenzoic acid (2.5 mg), catechin (1.5 mg), caffeic acid (3.3 mg), vanillin (3.0 mg), ferulic acid (1.5 mg), benzoic acid (4.1 mg), phloridzin (3.9 mg), cinnamic acid (2.7 mg), and phloretin (3.0 mg) were placed into a 10 mL volumetric flask, diluted with anhydrous methanol (chromatographic alcohol), and stored as the mother solution at a low temperature (4 °C) for subsequent use. The mixed standard sample was immediately prepared with 50 μL of each phenolic acid and 1 mL of methanol (chromatographic alcohol). The structures of 11 phenolic acids are shown in Figure S2.

### 4.4. Collection of Phenolic Acids Secreted by Roots

A total of 5 seedlings with similar growth outcomes were selected from the sand culture substrate by 5-point sampling, transferred to 250 mL of distilled water, pumped, and cultured at room temperature for 5–6 h under light, and the extract was collected. The extract was fully extracted with 200 mL of dichloromethane (analytical alcohol) 3 times, and the organic layers were combined, concentrated until dry in a rotary evaporator under reduced pressure, and reconstituted with 1.5 mL of methanol (chromatographic alcohol). The samples were placed in a refrigerator at −20 °C for 12 h to promote the precipitation of non-methanol-dissolved substances. After filtration through a 0.45 μm filter membrane, they were placed into 1.5 mL centrifuge tubes and stored in a refrigerator at −20 °C for subsequent use.

### 4.5. Effect of Leaching Solution on Seedlings

#### 4.5.1. Effect on Seedling Growth and Development

The growth of *M. hupehensis* Rehd. seedlings was estimated every 15 d after the seedlings had 6–7 true leaves. The plant height and ground diameter were measured using vernier calipers; the chlorophyll content was measured using a SPAD-502 portable chlorophyll meter. A desktop scanner was to digitize images of the root system (NUScan 700, Tai’an, China), and morphological measurements were obtained using image analysis software (Deta-T scan, Delta-T Devices Lad, Cambridge, UK). Chlorophyll fluorescence was measured using a basic modulation fluorometer (WALZ Co., Dalian, China), and the maximum photochemical efficiency of photosystem II (PSII) *F_v_/F_m_*= (*F_m_* − *_m_*/*F_m_*) was measured. The photochemical quenching coefficient was determined using the following formula: qP = (*F_m_′* − *F_s_*)/(*F_m_′* − *_m_′*). The non-photochemical quenching coefficient was determined using the following formula: NPQ = (*F_m_ − F_m_′*)/*F_m_′*.

#### 4.5.2. Determination of Enzyme Activity

The activities of SOD [41], POD [42], and CAT [43] and the content of MDA [44] were determined following the methods of Yoshioka et al.

### 4.6. Chromatographic Conditions

#### 4.6.1. Chromatographic Conditions for PAHs

The samples were analyzed by HPLC (UltiMate 3000 HPLC, Phenomenex EnviroSep-PP (125 mm× 4.6 mm) column) using the following conditions: column temperature 30 °C; injection volume 10 μL, mobile phase, acetonitrile, and water; gradient elution program, Table S1; UV detection, 254 nm; and fluorescence-detector wavelength program (Tables S2 and S3).

#### 4.6.2. Phenolic Acid Chromatographic Conditions

The phenolic acids in the samples were determined using HPLC (Waters 515 HPLC, Kromasil C18 (125 mm × 4.6 mm) column). The determination conditions were as follows: column temperature, 28 °C; injection volume 20 μL, mobile phase, acetonitrile, and water; gradient elution procedure, Table S4; and dual-wavelength UV detector (waters), 280 nm (Table S4).

### 4.7. Analysis Software

SPSS13.0 was used to test the significance of differences in each index between treatments; the levels of significance were *p* < 0.05 and *p* < 0.01. The LSD method was used for multiple comparisons.

The allelopathy effect sensitivity index (RI) was estimated using Williamson’s method [45]: RI = 1 − C/T (T ≥ C) or RI = T/C − 1 (T < C), where C is the control value and T is the treatment value. RI > 0 indicates a promoting effect and RI < 0 indicates an inhibiting effect. The absolute value of RI represents the intensity of allelopathy (autotoxicity).

## 5. Conclusions

Low-concentration root extract treatment (A1) significantly promoted the growth of *M. hupehensis* Rehd. seedlings, chlorophyll synthesis in leaves, the growth of seedling roots, and POD and CAT activities; inhibited the growth of ground diameter; and had no effect on SOD activity and fluorescence parameters. A6 had significant negative effects on all indices except the NPQ value.

Low-concentration soil extract treatment (B1) significantly promoted the growth of *M. hupehensis* Rehd. seedlings, chlorophyll synthesis in leaves, and the growth of seedling roots, and had no effect on the ground diameter; the activities of POD, SOD, and CAT; and fluorescence parameters. B5 and B6 increased NPQ, but had negative effects on all other indexes.

At the end of the treatment period, the root and soil extracts inhibited the secretion of gallic acid, p-hydroxybenzoic acid, benzoic acid, and phlorizin, but promoted the secretion of caffeic acid. Root extract treatment also inhibited catechin secretion and promoted the secretion of phloretin; soil extract treatment promoted the secretion of cinnamic acid. The secretion of the other phenolic acids varied depending on the concentration of leaching liquor applied.

## Data Availability

Data are presented in the article. All data and material are available from the manuscript.

## Supplementary Materials

The following supporting information can be downloaded at: https://www.mdpi.com/article/10.3390/plants11212968/s1, Table S1: Process diagram; Table S2: Gradient eluting procedure; Table S3: Fluorescence detector’s changes in procedures wavelength; Table S4: Gradient eluting procedure; Figure S1: 16 PAHs of EPA priority control; Figure S2: Structure of phenolics compounds; Figure S3: Standard sample 1 ((200 ng/g) in 254 nm ultraviolet detection), 2 ((20 ng/g) in different wavelengths fluorescence detection); Figure S4: Standard sample in 280 nm ultraviolet detection.

## Abbreviations

ARD: apple replant disease; *F_v_/F_m_*: PSII original light-energy-conversion efficiency; NPQ: non-photochemical quenching coefficient; qP: photochemical quenching coefficient; RI: indices of allelopathic effects; PAHs: polycyclic aromatic hydrocarbons; SOD: superoxide dismutase; POD: peroxidase; CAT: catalase; MDA: malondialdehyde.
