# Supplementary material for: Effects of Organic Acid Root Exudates of Malus hupehensis Rehd. Derived from Soil and Root Leaching Liquor from Orchards with Apple Replant Disease
Source: Plants (Basel). 2022 Nov 3;11(21):2968. doi: 10.3390/plants11212968 (PMC9654845; doi:10.3390/plants11212968)
Supplement: Supplementary file 1 [file plants-11-02968-s001.zip › plants-1947073-supplementary.pdf]

Supplementary material

Table S1. Process diagram

	Treatment						
	CK	Low			High		
root：water	A0	A1	A2	A3	A4	A5	A6
	0:1	1:100	1:40	1:20	1:10	1:2	1:1
soil：water	B0	B1	B2	B3	B4	B5	B6
	0:1	1:100	1:40	1:20	1:10	1:2	1:1

Note: Named the root extract A, the soil extract B, and the low concentration A1-A3, B1-B3; High concentrations for A4-A6, B4-B6.

Table S2. Gradient eluting procedure

Time (min)	A% Water	B% Acetonitrile
-5.0	60	40
0	60	40
2	60	40
22	0	100
32	0	100
32.1	60	40

Table S3. Fluorescence detector's changes in procedures wavelength

Time (min)	Excitation wavelength(nm)	Emission wavelength(nm)
0.00	256	390
14.80	275	420
17.00	270	385
21.00	290	430
25.90	305	480
32.00	256	390

Table S4. Gradient eluting procedure

Time(min)	A% Water	B% Acetonitrile
0	90	10
30	65	35
40	65	35
42	90	10
45	90	10

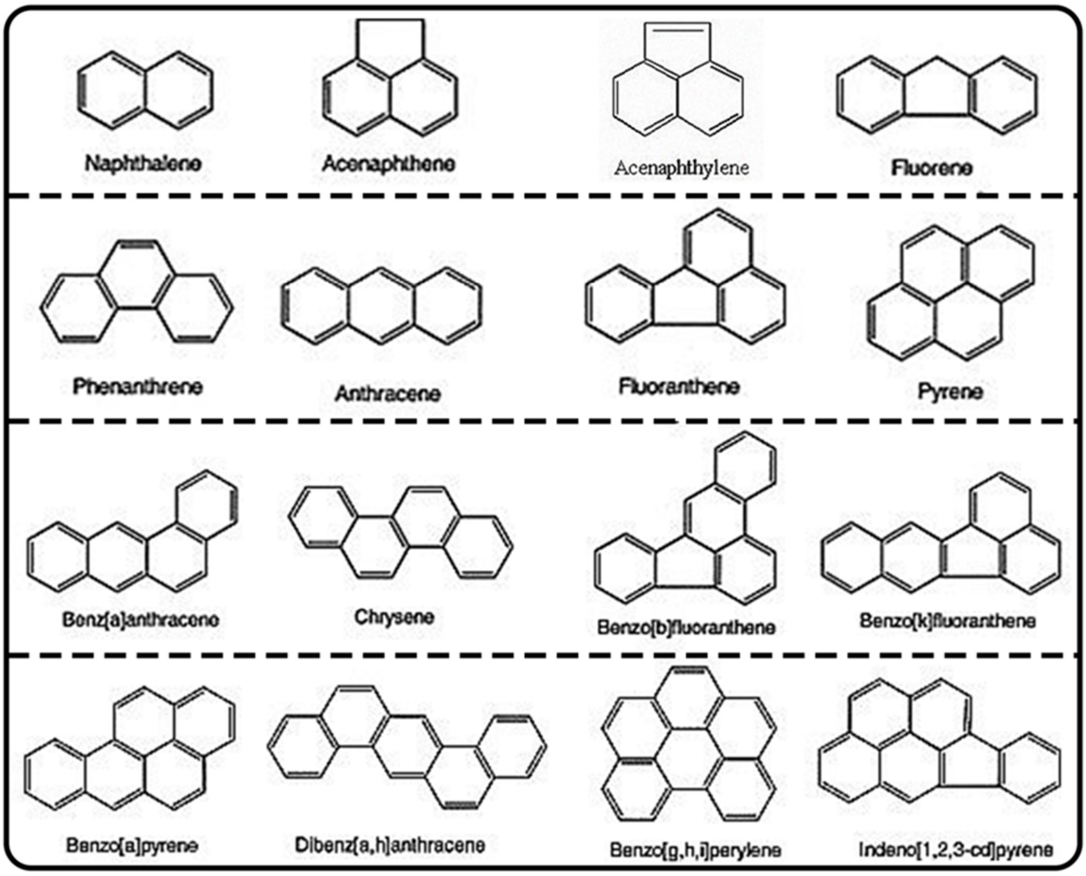

Figure S1. 16 PAHs of EPA priority control

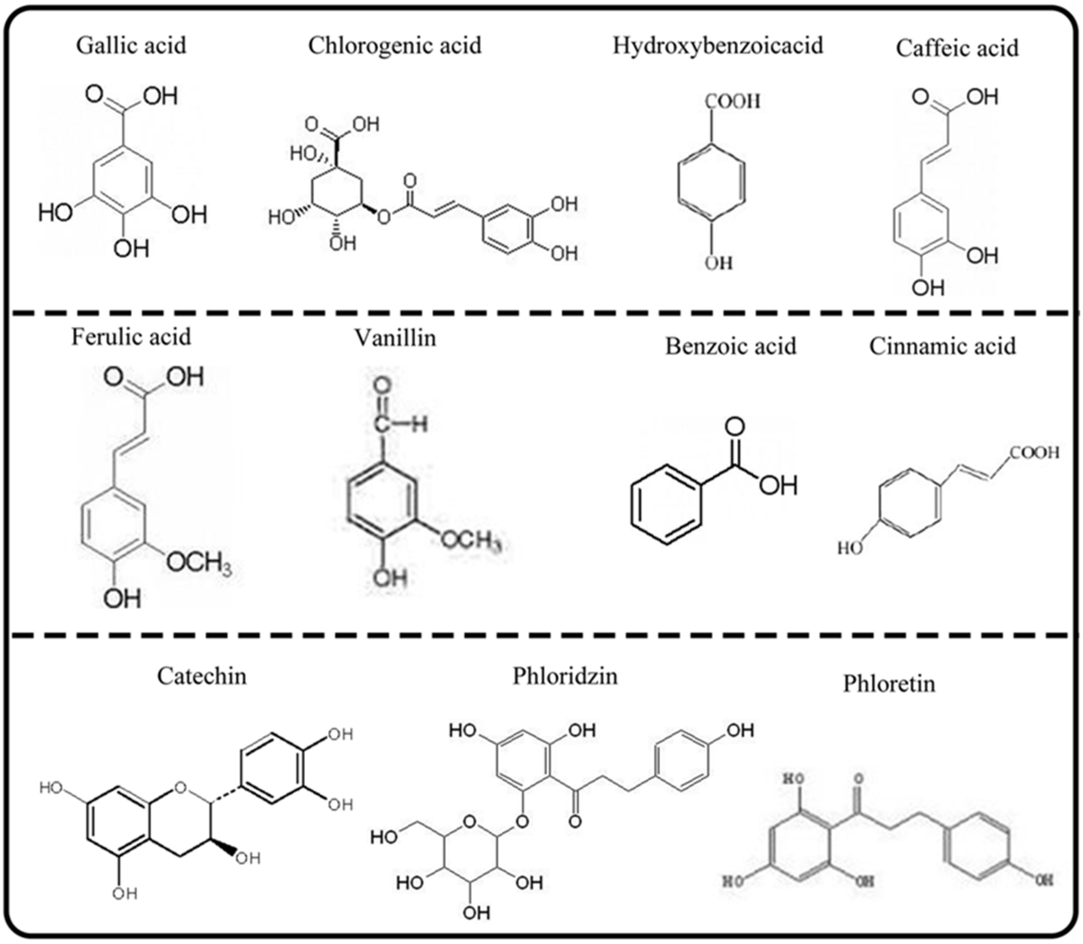

Figure S2. Structure of phenolics compounds

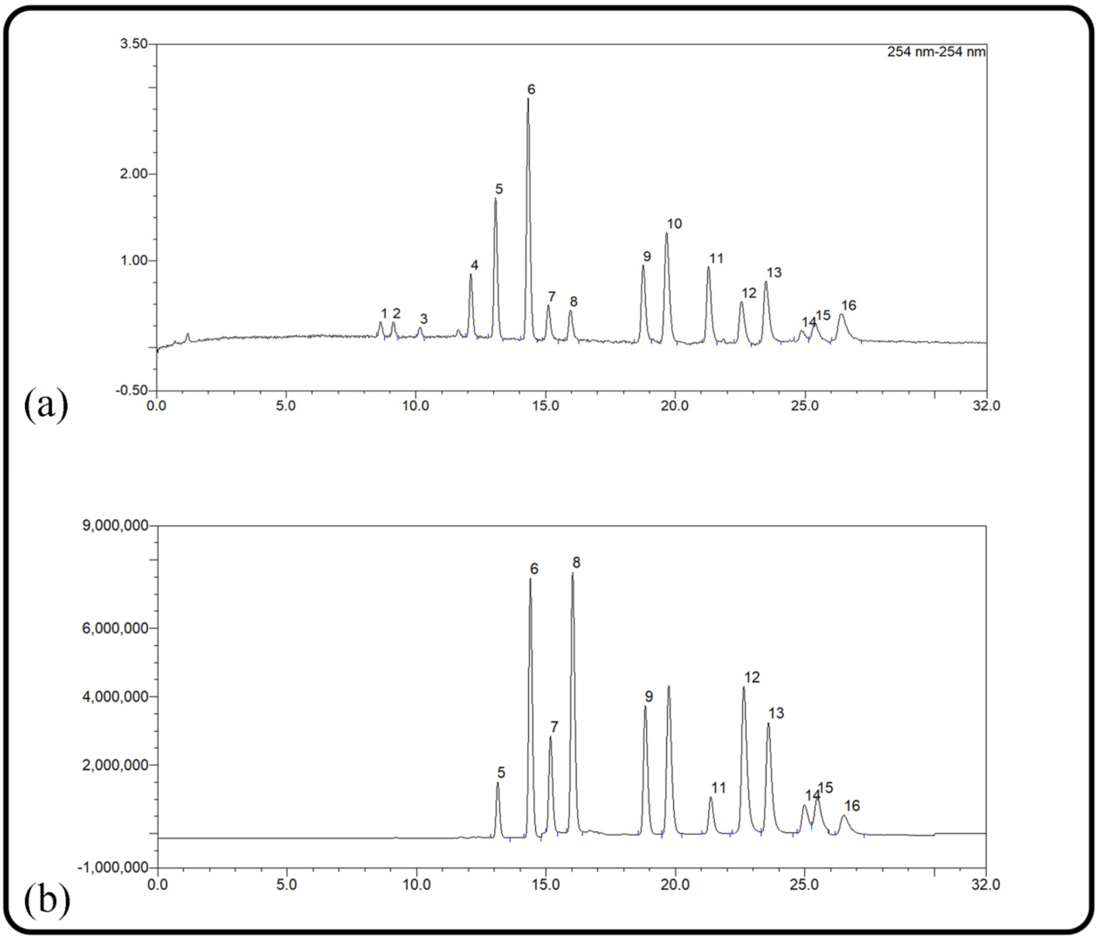

Figure S3. Standard sample 1 ((200 ng/g) in 254nm ultraviolet detection)、2 ((20 ng/g) in different wavelengths fluorescence detection)

1-Nap，2-Ace，3-Acy,4-Flu，5-Phe，6-Ant，7-Flt，8-Pyr，9-Baa，10-Chr，11-Bbf，12-Bkf，13-Bap，14-Daa，15-Bpe，16-Ilp

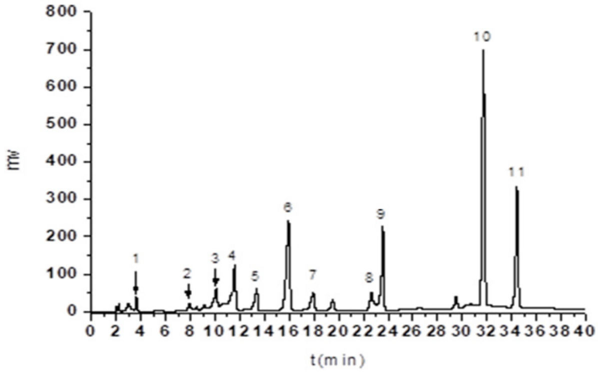

Figure.S4 Standard sample in 280nm ultraviolet detection

1-Gallic acid，2-Chlorogenic acid，3-Hydroxybenzoic acid，4-Catechin，5-Caffeic acid，6- Ferulic acid，7-Vanillin，8-Benzoic acid，9- Phloridzin，10-Cinnamic acid，11- Phloretin
